# Interaction of BCI with the underlying neurological conditions in patients: pros and cons

**DOI:** 10.3389/fneng.2014.00042

**Published:** 2014-11-18

**Authors:** Aleksandra Vuckovic, Jaime A. Pineda, Kristen LaMarca, Disha Gupta, Christoph Guger

**Affiliations:** ^1^School of Engineering, University of GlasgowGlasgow, UK; ^2^Cognitive Science Department, University of CaliforniaSan Diego, La Jolla, CA, USA; ^3^Clinical Psyhology, California School of Professional PsychologySan Diego, CA, USA; ^4^Burke Rehabilitation Center, Burke-Cornell Medical Research InstituteWhite Plains, NY, USA; ^5^Guger Technologies OG, g.tec medical engineering GmbHGraz, Austria

**Keywords:** BCI performance, patients, amyothopic lateral sclerosys, spinal cord injury, autism spectrum disorders, stroke volume, cerebral palsy

The primary purpose of clinical Brain Computer Interface (BCI) systems is to help patients communicate with their environment or to aid in their recovery. BCI can be used to replace, restore, enhance, supplement, or improve natural Central Neural System (CNS) output (Wolpaw and Wolpaw, 2012).

A common denominator for all BCI patient groups is that they suffer from a neurological deficit. As a consequence, BCI systems in clinical and research settings operate with control signals (brain waves) that could be substantially altered compared to brain waves of able-bodied individuals. Most BCI systems are built and tested on able-bodied individuals, being insufficiently robust for clinical applications. The main reason for this is a lack of systematic analysis on how different neurological problems affect the BCI performance.

This special issue highlights interaction of BCI systems with the underlying neurological problems and how performance of these BCI system differ compared to similar systems tested on healthy individuals. The issue presents 4 reviews (Friedrich et al., 2014; Pineda et al., 2014; Priftis, 2014; Rupp, 2014) and 8 experimental studies (Ang et al., 2014; Daly et al., 2014; Ono et al., 2014; Song et al., 2014; Xu et al., 2014; Young et al., 2014a,b,c). It covers studies on five different patient groups: stroke (Ang et al., 2014; Ono et al., 2014; Song et al., 2014; Young et al., 2014a,b,c), spinal cord injury (SCI) (Rupp, 2014; Xu et al., 2014), autism (Friedrich et al., 2014; Pineda et al., 2014), cerebral palsy (CP) (Daly et al., 2014) and amyotrophic lateral sclerosis (ALS) (Priftis, 2014). Three different types of BCI are presented: motor imagery, P300 and neurofeedback (operant conditioning). In the presented papers, BCI has been used either on its own or in a combination with an external device such as a robot or a functional electrical stimulation (FES).

Review papers discuss several possible applications of BCI including methods to replace (Priftis, 2014; Rupp, 2014), restore (Rupp, 2014) and improve (Friedrich et al., 2014; Pineda et al., 2014; Rupp, 2014) natural CNP output. Several experimental studies in this special issue present BCI applications to improve and restore CNP functions (Ang et al., 2014; Ono et al., 2014; Young et al., 2014a,b) while some present basic research papers looking into the effect of BCI training on the cortical activity (Song et al., 2014; Young et al., 2014b,c) or exploring EEG signature characteristic for a certain patient group, such as SCI or CP (Daly et al., 2014; Xu et al., 2014).

In two review articles Pineda et al. and Friedrich et al. look into the application of BCI on a relatively novel group of patients, autistic children, who show deficits in social and communicative skills, including imitation, empathy, and shared attention, as well as restricted interests and repetitive patterns of behaviors. They discuss evidences for model-based neurofeedback approach for treating autism and propose a BCI game for treating both high and low functioning autistic patients. The game is unique in that it includes social interactions and provides neural- and body-based feedback that corresponds directly to the underlying significance of the trained signals as well as to the behavior that is reinforced. A review by Rupp provides a comprehensive critical analysis of pros- and cons- of different types of BCI for spinal cord injured patients. He also discusses advantages and disadvantages of using BCI for communication, wheelchair and environmental control, control of neuroprosthesis and for clinical, rehabilitation purposes. This paper provides a valuable analysis of different medical and personal factors which might affect the performance of a BCI. While some of these factors are specific for spinal cord injured patients, many of them would exist in most patient groups using BCI. A review paper by Priftis provides a critical analysis of the evidences of the effectiveness of P300 speller as a communication tool for ALS patients. This is one of the rare application for which a commercially available solution exists (intendix, g.tec medical engineering GmbH, g.USBamp P300 model, Guger Technologies OG, Austria). While accuracy of this type of BCI reaches 90% in able-bodied, only 70% can be achieved in patients (Ortner et al., 2011). Priftis (2014) therefore concluded that requirements of ALS patients haven't been met yet, and highlights a striking fact that a tiny portion of published papers on P300 BCI presents experimental studies on ALS patients.

Papers showing experimental results in the special issue are either oriented toward rehabilitation or toward a basic science research. Stroke remains the most frequently tested patient population. In a randomized controlled trial on 21 chronic stroke patients, Ang et al. compare three hand and arm rehabilitation therapies, BCI with a haptic knob (HK) robot, HK alone or a standard physiotherapy. They provided evidences for BCI-HK group achieving significantly larger motor gain than the other two groups.

Ono et al. combined motor imagery based BCI with two different types of feedback for rehabilitation of hand function in chronic stroke patients; a visual and somatosensory. While both feedback modalities increased cortical response, as measured by the intensity of event-related desynchronization (ERD), only BCI training with somatosensory feedback provided improved motor function. This paper therefore demonstrates that changes in the cortical level might not necessarily be indicators of functional recovery.

An interesting case study by Young et al. (2014a), which fits well with the topic of the special issue, investigated how the preexisting neurological condition (congenital deafness) of a stroke patient influences performance of BCI system used for motor rehabilitation. The same research group provided a comprehensive analysis on the influence of BCI training on functional brain connectivity and brain organization, as measured by EEG and fMRI and it's relation to motor gains (Song et al., 2014; Young et al., 2014b,c). In a controlled study on 14 chronic hemiplegic patients they showed that only a treatment group, which received BCI-FES therapy, showed differential changes in brain activity patterns between lesioned and non lesioned hemisphere, which were associated with changes in a specific motor function (Young et al., 2014b). Using diffusion tensor imagining they showed that baseline fractional anisotropy of the posterior limb of the internal capsule predicts motor recovery (Song et al., 2014). They also used fMRI to measure brain activity in stroke patients in a simple tapping task before and after a BCI intervention, showing that task-based functional connectivity correlates with gain in the motor outcome. However they also gave a word of warning indicating that BCI therapy might produce both adaptive and maladaptive changes (Young et al., 2014c).

Xu et al. compared movement related cortical potentials (MRCP) between three groups: able bodied volunteers, chronic paraplegic patients with central neuropathic pain and chronic paraplegic patients with no pain. They found significantly larger MRCP in both paraplegic patients groups compared to able-bodied people, independent on the underlying sensory loss or presence of chronic pain. This contrasts studies based on ERD analysis, in which paralysis and pain showed differential effect on the activity of the sensory-motor cortex (Vuckovic et al., 2014) and in which paraplegic patients with no pain have weaker ERD signatures than able-bodied people (Pfurtscheller et al., 2009; Vuckovic et al., 2014); the study indicates that in this patient group, for motor imagery based BCI, time and phase locked MRCP might be a better suited feature than time but not phase locked ERD.

Daly et al. provided one of the rare BCI studies on adults with CP. They showed that motor imagery in patients with CP results in significantly less ERD and less functional connectivity compared to the able-bodied, indicating potentially lower BCI performances.

In summary, for BCIs it is still a long way to presenting an adequate replacement of the existing technologies for communication and control in patients with a minimum of preserved motor and cognitive function. Rehabilitation seems to be the area which provides the most immediate measure of benefit to a user. Rehabilitation is limited to a certain period of time and is typically performed in clinical environment, therefore can be operated by a clinically trained person. Recent tendencies to prolonged, home based rehabilitation will however likely increase requirements for a rehabilitation BCI in respect to size, price, esthetic, and user friendliness.

We are optimistic that this special issue will generate a body of knowledge valuable both to researchers working with clinical populations, but also to a vast majority of BCI researchers testing new algorithms on able-bodied people. This should lead toward more robust or tailor-made BCI protocols, facilitating translation of research from laboratories to the end users.

## Conflict of interest statement

The authors declare that the research was conducted in the absence of any commercial or financial relationships that could be construed as a potential conflict of interest.
